# A Consensus Method for the Prediction of ‘Aggregation-Prone’ Peptides in Globular Proteins

**DOI:** 10.1371/journal.pone.0054175

**Published:** 2013-01-10

**Authors:** Antonios C. Tsolis, Nikos C. Papandreou, Vassiliki A. Iconomidou, Stavros J. Hamodrakas

**Affiliations:** Department of Cell Biology and Biophysics, Faculty of Biology, University of Athens, Panepistimiopolis, Athens, Greece; Deutsches Zentrum für Neurodegenerative Erkrankungen e.V., Germany

## Abstract

The purpose of this work was to construct a consensus prediction algorithm of ‘aggregation-prone’ peptides in globular proteins, combining existing tools. This allows comparison of the different algorithms and the production of more objective and accurate results. Eleven (11) individual methods are combined and produce AMYLPRED2, a publicly, freely available web tool to academic users (http://biophysics.biol.uoa.gr/AMYLPRED2), for the consensus prediction of amyloidogenic determinants/‘aggregation-prone’ peptides in proteins, from sequence alone. The performance of AMYLPRED2 indicates that it functions better than individual aggregation-prediction algorithms, as perhaps expected. AMYLPRED2 is a useful tool for identifying amyloid-forming regions in proteins that are associated with several conformational diseases, called amyloidoses, such as Altzheimer's, Parkinson's, prion diseases and type II diabetes. It may also be useful for understanding the properties of protein folding and misfolding and for helping to the control of protein aggregation/solubility in biotechnology (recombinant proteins forming bacterial inclusion bodies) and biotherapeutics (monoclonal antibodies and biopharmaceutical proteins).

## Introduction

Protein and peptides may form aggregates under various conditions [1]. These aggregates may lack any ordered structure or may be characterized by different degrees of order. Amyloid structures constitute a specific subset of insoluble fibrous protein aggregates. These structures arise by sequences that allow the formation of intermolecular beta-sheet arrangements and their packing in the highly stable three-dimensional structure of amyloid fibrils [2]–[4]. The biological properties of these cross-β fibrillar aggregates differ from those of amorphous aggregates. Amyloid fibrils have also functional roles throughout all kingdoms of life as protective formations, structural scaffolds, water tension modulators, adhesives *etc* [5]–[7]. Furthermore, protein deposits found in association with many human diseases are often characterized by amyloid structure. These ‘conformational diseases’ are collectively called amyloidoses and can be systematic or localized (affecting only specific organs). They include, among others, neurodegenerative diseases (e.g. Alzheimer's, Huntington's, Parkinson's), type II diabetes and prion diseases [8], [9].

Many studies suggest that not all regions of a polypeptide chain are equally important for determining its aggregation tendency. It seems that protein aggregation is mediated by short ‘aggregation prone’ peptide segments [10]–[12]. These aggregation nucleating regions can be detected in the sequence of a protein utilizing bioinformatics prediction methods, based on physicochemical principles (*phenomenological models*) or/and molecular simulation approaches [13].

Many prediction algorithms have been developed during the last decade to perform this task. Kallberg *et al*. [14] searched for α-helices with a tendency to form β-sheets. Yoon and Welsh [15], [16] calculated the hidden β-propensity to find regions that appear to be natively α-helical but have nonetheless the ability to form β-strands. Hamodrakas *et al*. [17] have similarly looked for “conformational switches” in sequences, *i.e*. regions with a high predicted tendency to form both α-helices and β-strands, using the consensus secondary structure prediction program SecStr [18].

Dobson and colleagues [19], [20] made the first efforts to predict the effects of mutations on peptide/protein aggregation rate and later, Tartaglia *et al*. [21], [22] also studied the factors that determine the aggregation rate of proteins. Lopez de la Paz and Serrano [23] identified a sequence pattern that is involved in the formation of amyloid-like fibril, using saturation scanning mutagenesis analysis on the *de novo*-designed amyloidogenic peptide STVIIE.

Fernandez-Escamilla *et al*. [24] developed a statistical mechanics algorithm (TANGO) designed to predict β-sheet aggregation of proteins, which is different from amyloid fibril formation tendency but is highly correlated. Idicula-Thomas and Balaji [25] tried to understand the sequence characteristics (including aliphaticity, instability, orderliness and sheet propensity) of proteins that are prone to form amyloid fibrils.

Thompson *et al*. [26] and Zhang *et al*. [27] identified computationally peptide segments that fit as β-strands in a stacked β-sheet structure based on the solved microcrystal structures obtained from the peptides GNNQQNY and NNQQNY [28], known amyloidogenic regions from the yeast prion Sup35. Saiki *et al*. [29] developed a prediction method based on a structural model of amyloid fibrils.

Galzitskaya *et al*. [30] related the Average Packing Density of stretches of residues to the formation of amyloid fibrils. Later, they improved this method including hydrogen bonding interactions (FOLDAMYLOID) [31]. Zibaee *et al.* [32] developed SALSA (Simple ALgorithm for Sliding Averages) to locate regions with high propensity for β-strand structure. AGGRESCAN from Conchillo-Solé *et al*. [33] was based on an aggregation propensity scale for natural amino acids derived from *in vivo* experiments. Trovato *et al*. [34] (PASTA) and Bryan *et al*. [35] (BETASCAN), looked for potential β-strand pairs. Clarke and Parker [36] combined a coarse-grained physico-chemical protein model with a highly efficient Monte Carlo sampling technique to identify amyloidogenic sequences.

Tian *et al*. [37] developed a phenomenological method (Pafig) based on Support Vector Machines (SVM), for the identification of hexapeptides associated with amyloid fibrillar aggregates. 41 physicochemical properties were selected by a two-round selection from 531 physicochemical properties in the Amino acid index database (AAindex). Recently, Nair *et al*. [38] published a paper in which they described the combination of SVMs with ANNs for the identification of amyloidogenic peptides.

WALTZ from Maurer-Stroh *et al*. [39] used position-specific scoring matrices to determine amyloid-forming sequences. David *et al*. [40] used a naive Bayesian classifier and a weighted decision tree for predicting the amyloidogenicity of immunoglobulin sequences. O'Donnell *et al*. [41] designed AmyloidMutants to predict the structural and mutational landscapes of amyloid fibrils using energy calculations.

Our lab developed a consensus algorithm for the prediction of amyloidogenic determinants from sequence alone, called AMYLPRED [42] (2009). AMYPLRED was based on 5 different methods. We found that its results tend to be slightly more accurate than the individual predictors. We have improved this tool recently, creating AMYLPRED2, by adding 6 novel, recently published, algorithms. Therefore, AMYLPRED2 combines 11 different methods in total.

In this work, we apply AMYLPRED2 on a set of 33 amyloidogenic proteins, showing that it performs better than its subordinate methods, we indicate how it can be used to improve the solubility of recombinant proteins, inhibiting the formation of bacterial inclusion bodies, and we provide a specific example of its possible use for the production of more soluble humanized monoclonal antibodies, in biotherapeutics.

## Methods

The consensus web tool AMYLPRED2 (available at http://biophysics.biol.uoa.gr/AMYLPRED2/) includes the following methods: Aggrescan [33], AmyloidMutants [41], Amyloidogenic Pattern [23], Average Packing Density [30], Beta-strand contiguity [32], Hexapeptide Conformational Energy [27], NetCSSP [16], Pafig [37], SecStr (Possible Conformational Switches) [17], Tango [24] and Waltz [39]. AMYLPRED2 takes the results of Amyloidogenic Pattern, Average Packing Density, Beta-strand contiguity, Hexapeptide Conformational Energy and SecStr from the output of the original AMYLPRED, which calls the individual scripts locally in our server. The β-strand contiguity script (which was written by our lab), and the Pafig script are also executed locally. The output of the rest of the methods is taken directly from their own respective servers.

The consensus of these methods is defined as the hit overlap of at least n/2 (rounded down) out of n selected methods (i.e. 5 out of 11 methods, if the user chooses to use all available methods). This is an empirical threshold that was chosen based on many tests we performed. We ran multiple subsets of proteins with multiple combinations of those 11 algorithms and with all possible thresholds (2–11). The lower the threshold was, the higher the sensitivity (the lower the specificity) and vice versa. In most cases, the best balance between sensitivity and specificity (best Q and MCC) was provided by the hit overlap of at least n/2 (rounded down) out of n methods.

The primary output of the program is the consensus prediction. However, the individual predictions of the incorporated methods are also made available by pressing the button “Show/hide methods”. Furthermore, a consensus histogram is shown by pressing the button “Show/hide consensus”. All results are also made available in the form of a text file, maintained on the server for one day (24 hours). These features allow a researcher to compare individual predictions, evaluate the results and focus on the predicted segments of interest. For example, a consensus prediction from 10 out of 11 methods for an amyloidogenic segment is way stronger from a consensus prediction in which only 5 methods agree. Nevertheless, consensus agreement lower than the threshold of 5 may reveal hidden amyloidogenic segments, which may play an important role in the amyloidogenic process. It is up to the researcher to use/evaluate the results based on other available data and experience.

Many individual methods provide several different settings. We tried them with many different values and combinations of values. Finally, we chose those values that yield the best performance, for each method alone, based on the results of tests that the individual authors provide in their published papers and -in addition- we performed our own tests with multiple subsets of amyloidogenic proteins. For AmyloidMutants, we use the default settings and the cross-beta pleat (serpentine) structural scheme (the other structural schemes had some performance issues and they often failed to give any results). For Average Packing Density, values above 21.4 [30], obtained from a five-residue long sliding window are considered as hits. For Beta-strand contiguity, we use a threshold value of MβP > = 1.2 [32] and we consider total y values above 20 as hits. For Hexapeptide Conformational Energy, energy values below −27.00 [27] are considered as hits. For NetCSSP, we use the dual network architecture as it has greater accuracy [16]. The amyloidogenic hidden beta propensity (HβP) is calculated using the form HβP  =  P(beta)/P(helix). Residues with values of HβP above 1 and of P(beta) above 6 are considered as hits. For Pafig, we use a threshold for the Reliability Index of 7 [37]. For Tango, Tango 2.1 is used and scores above 5.00% for beta aggregation are considered as hits [24]. Tango requires a set of environmental parameters for each submission. The default values from the TANGO online submission form are used. For Waltz, we use pH  = 7.0 and a threshold value of 79.0 (High Sensitivity), because, according to our own tests, it gives better overall results [43]. We have to note that the default setting ‘Best overall performace’ of Waltz (threshold 92.0) had lower Q ( = 56.20) and MCC ( = 0.157) for the test set of 33 proteins. Some of the individual methods have limitations regarding the minimum length of the input sequence. Pafig needs at least 6 residues, NetCSSP at least 7 residues, whereas AmyloidMutants needs a minimum of 20 residues. For more details regarding how the individual methods are used in the consensus prediction, please consult AMYLPRED2 web help page in this URL: http://biophysics.biol.uoa.gr/AMYLPRED2/.

We have tested the consensus method of AMYLPRED2 against each of the individual methods on a set of 33 amyloidogenic proteins for which experimental data is available (Table S1). For this test set, we collected from the literature, as many as we could, well-studied amyloidogenic proteins. We took care to cross-check the amyloidogenic regions of these proteins. To make sure that our test will not yield ‘artificial’ and/or unreliable results, we searched to find data from many published experiments and different experimental methods that support the amyloidogenicity of these specific regions. We excluded two proteins, Laminin alpha-1 chain of mouse (3080 AA) and Human complement receptor type 1 (2039 AA), because they are huge and only a very small segment of them has been studied and this would introduce bias to the results. We also excluded proteins with similar sequences to avoid redundancy (e.g. we included, in the test set, only the human Major Prion Protein and not that of the mouse because of the similarity between the sequences). Because the number of proteins that form amyloid fibrils is relative small, we didn't exclude the proteins that some individual methods used for data collection and/or training. For example, the training set of Pafig is so large that if we had to remove these proteins, we wouldn't have many left (We must note that Pafig has performed its own cross-validation test [37]).

Amino acid sequences of the proteins used in this study were retrieved from UniprotKB (http://www.uniprot.org) [44]. Protein structures were retrieved from PDB (http://www.pdb.org) [45]. For each protein structure used, residues accessible to the solvent or buried into a protein's hydrophobic interior were determined utilizing the algorithm DSSP [46]. True/false positives (TP, FP) and true/false negatives (TN, FN) for each method were counted on a per residue basis. Sensitivity is measured as TP/(TP + FN), specificity as TN/(TN + FP), Q is calculated as (Sensitivity + Specificity)/2 and Matthews Correlation Coefficient (MCC) as (TP * TN – FP * FN)/√((TN + FN) * (TN + FP) * (TP + FN) * (TP + FP)).

## Results

We have found that AMYLPRED2 has the best Q and MCC compared to its subordinate methods (Table 1). Beta-strand contiguity and Pafig are following in the next positions. AMYLPRED2 has almost the same specificity with the original AMYLPRED, but there is a 6% increase in its sensitivity.

**Table 1:** Performance of the tool AMYLPRED2 and of its subordinate methods, on a set of 33 amyloidogenic proteins (see Table S1).

METHOD	TP	TN	FP	FN	SENSIIVITY (%)	SPECIFICITY (%)	Q (%)	MCC
Aggrescan [33]	445	5210	1363	813	35.37	79.26	57.32	0.13
AmyloidMutants [41]	524	4924	1649	734	41.65	74.91	58.28	0.14
Amyloidogenic Pattern [23]	176	6208	365	1082	13.99	94.45	54.22	0.12
Average Packing Density [30]	361	5529	1044	897	28.70	84.12	56.41	0.12
Beta-strand contiguity [32]	417	5628	945	841	33.15	85.62	59.39	0.18
Hexapeptide Conf. Energy [27]	494	5172	1401	764	39.27	78.69	58.98	0.15
NetCSSP [16]	645	4287	2286	613	51.27	65.22	58.25	0.12
Pafig [37]	651	4695	1878	607	51.75	71.43	61.59	0.18
SecStr [17]	143	6205	368	1115	11.37	94.40	52.88	0.09
Tango [24]	172	6282	291	1086	13.67	95.57	54.62	0.14
Waltz [39]	710	4300	2273	548	56.44	65.42	60.93	0.16
AMYLPRED [42]	415	5668	905	843	32.99	86.23	59.61	0.19
AMYLPRED2	494	5553	1020	764	39.27	84.48	61.88	0.22

True/false positives (TP, FP) and true/false negatives (TN, FN) for each method were counted on a per residue basis. Sensitivity is measured as TP/(TP + FN), specificity as TN/(TN + FP), Q is calculated as (Sensitivity + Specificity)/2 and Matthews Correlation Coefficient (MCC) as (TP * TN – FP * FN)/√((TN + FN) * (TN + FP) * (TP + FN) * (TP + FP)).

We should note that the numbers shown in Table 1 are subject to change as more experimental data become available. Regions currently regarded as non-amyloidogenic are not necessarily so and may prove to be in fact amyloidogenic in the future. Predictions with a strong agreement among many different methods may suggest amyloidogenic determinants/‘aggregation-prone’ sequences currently unknown and consensus methods like AMYLPRED2 might therefore provide valuable hints to researchers.

Indeed, our lab has synthesized peptides, representative for several regions (more than 25) that AMYLPRED has indicated as amyloidogenic in proteins related to amyloidoses and we have found using Electron Microscopy, X-ray diffraction, Congo Red staining and ATR FT-IR and Raman spectroscopy that these peptides, indeed, form amyloid fibrils *in vitro* (In preparation, see also ref. 45).

In Table S2, we have calculated the MCC per protein per method. This allows us to examine some performance details. We see that many methods fail in specific proteins. For example, most methods have a low MCC with regard to some large proteins (e.g. Gelsolin, Kerato-epithilin, Lactoferrin). The main reason for that is the fact that only a relative small portion of them have been studied and confirmed experimentally to be amyloidogenic. Therefore, there are too many false(?) positives for the rest of these proteins.

We also see that most methods have problems with some prion proteins from fungi like Sup35, Ure2 and Het-s (Sup35 and Ure2 are Q/N-rich proteins). But they seem to predict quite well the amyloidogenicity of the human Major prion protein.

With the exception of Waltz, most methods predict different regions from the experimentally verified for Calcitonin (a 32-amino acid peptide hormone). They also seem to perform poorly for bacterial Cold Shock Protein from *Bacillus subtilis*, a small, amyloidogenic, protein (They predict only a small segment as amyloidogenic, so there are many false negatives).

In Table S2, we also see the average values of MCC per protein per method. In this calculation, every protein has the same weight (1/33) in the final result. This corrects some bias that originates from the size of proteins and the relevant problems we have discussed earlier. These results confirm that AMYLPRED2 has the best overall performance with a value of MCC equals to 0.34, while Waltz, AMYLPRED, Beta-strand contiguity and Aggrescan have values 0.29–0.27.

## Discussion

Amyloidoses are conformational diseases that affect an increasing number of individuals, deteriorating their quality of life and imposing, frequently, a major burden in their families. Many research groups around the world are trying to detect the causative agents and to discover a therapy against these diseases. Bioinformatics plays a key role towards the accomplishment of these efforts. Even though, prediction tools cannot substitute experimental work, they may help researchers in focusing at potential amyloidogenic regions for further experimental studies [47].

Another interesting application of the methods predicting ‘aggregation-prone’ sequences/amyloidogenic determinants is their use in the field of biotechnology. The recombinant proteins produced in bacterial cells often tend to aggregate, forming inclusion bodies [48]. This kind of protein aggregation has been shown to resemble amyloid fibril formation, and, actually, it has been shown conclusively that inclusion bodies contain amyloid fibrils [49]–[54]. There is clearly the possibility to improve the solubility of recombinant proteins by locating and altering the potential of the amyloidogenic determinants/‘aggregation-prone’ sequences [43].

Protein aggregation has also been connected to increased immunogenicity and undesirable immunogenic reactions [55]. Aggregation and immunogenicity constitute major bottlenecks during the discovery and development stages of biotherapeutics. It has been shown that ‘aggregation-prone’ regions predicted by AMYLPRED may overlap with immune epitopes in biotherapeutics (IFN-β), i.e. with regions that are responsible for immunogenicity [56].

**Figure 1:**
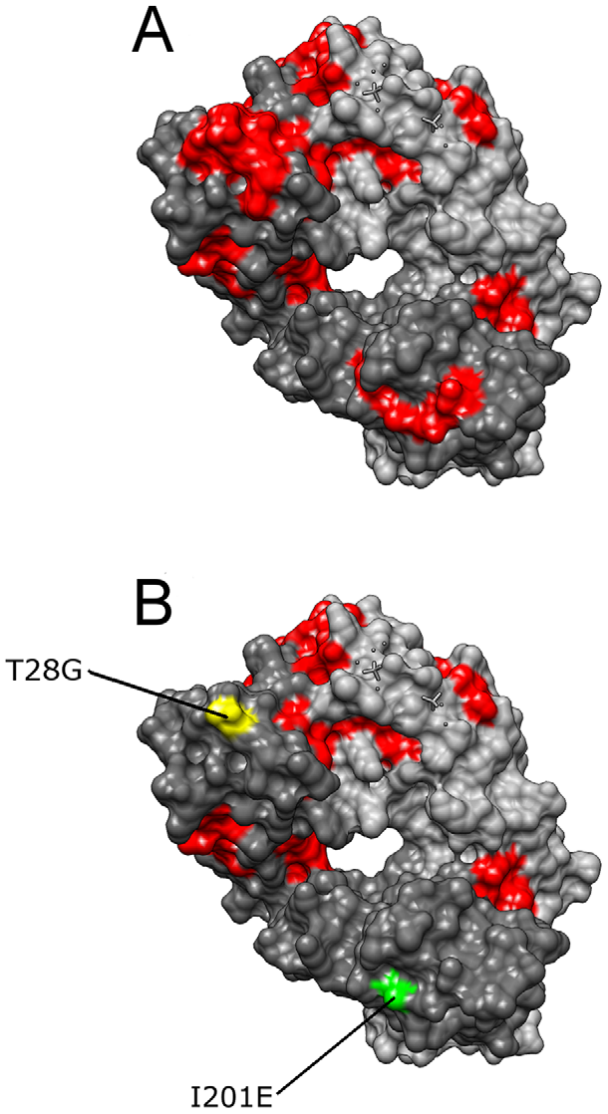
The crystal structure (space-filling model) of the anti-ErbB2 Fab2C4 (PDB code: 1L7I) is shown. (A). This is a humanized monoclonal antibody fragment that binds to the extracellular domain of the human oncogene product ErbB2 (ErbB2 has been shown to play an important role in the pathogenesis of certain aggressive types of breast cancer). Computationally predicted ‘aggregation-prone’ regions by AMYLPRED2 are coloured red. Performing only two single amino acid substitutions (T28G and I201E), the AMYLPRED2 output suggests that the antibody has ‘lost’ two crucial ‘aggregation-prone’ regions and may, therefore, be more soluble, not forming aggregates (B). Molecular graphics were performed with the UCSF Chimera package. Chimera, developed by the Resource for Biocomputing, Visualization, and Informatics at the University of California, San Francisco (supported by NIGMS 9P41GM103311) [60].

Furthermore, methods that are included in AMYLPRED2, have been used for the prediction of potential ‘aggregation-prone’ regions in commercial monoclonal antibodies and the discovery of ‘aggregation-prone’ motifs in biopharmaceuticals (albumin, insulin, factor VIII and others) [57], [58]. We provide an illustrated example of this use of AMYLPRED2 in Fig. 1, where the high resolution (1.80Å) crystal structure of the anti-ErbB2 Fab2C4 [59] (PDB code: 1L7I) is depicted, as a space-filling model (Fig. 1a) [60]. This is a humanized monoclonal antibody fragment that binds to the extracellular domain of the human oncogene product ErbB2. ErbB2 has been shown to play an important role in the pathogenesis of certain aggressive types of breast cancer. Amyloidogenic/‘aggregation-prone’ regions of anti-ErbB2, computationally predicted by AMYLPRED2, are coloured red. Performing only two single amino acid substitutions (T28G and I201E), the AMYLPRED2 output (Fig. 1b) suggests that the antibody has ‘lost’ two crucial ‘aggregation-prone’ regions and may, therefore, be more soluble, not forming aggregates that complicate drug development and therapy.

So, methods like AMYLPRED2 may help the researchers not only to find new therapeutic approaches against amyloidoses or to improve the existing ones, but, also, to design better drugs with fewer side effects (*Quality by Design*).

## Conclusions

Protein aggregation and amyloid fibril formation prediction methods might be used for screening therapeutic approaches against amyloidoses and the control and fine-tuning of protein solubility in the field of biotechnology. Furthermore, they may be used to improve protein solubility in biotherapeutics. Last but not least, these methods may improve our understanding of amyloid-fibril formation pathways/processes. A comparative and consensus tool, like AMYLPRED2, may help by offering more objective results and a direct comparison of existing methods and algorithms.

## Supporting Information

Table S1 **Prediction of amyloidogenic regions or “aggregation-prone” stretches, for 33 amyloidogenic proteins by AMYLPRED and AMYLPRED2, for comparison. Superscripts after each protein name (first column) refer to the relevant literature used (given at the bottom of the Table) to obtain experimental information.** The residue numbering for the sequence features (first column) refers to the respective Uniprot entries. The sequences of the mature proteins are given in the second column. Experimentally verified amyloid forming regions/“aggregation-prone” stretches are shown in bold. The residue numbering for the experimental and predicted regions (remaining columns) refers to the mature protein only. Bold font highlights hits that are in agreement with experimental data. Surface accessibility for these peptides was calculated in Å^2^, using DSSP, with a probe radius of 1.4 Å (which approximates the radius of a water molecule). One asterisk (*) denotes peptides on the surface of the relevant proteins using a per-residue cut-off of 20 Å^2^ (corresponding to ∼2 water molecules per residue). A double asterisk (**) denotes semi-surface peptides (with a per-residue value between 10–20 Å^2^).
(PDF)

Table S2 **MCC per protein per method.** The main reason that the majority of methods has a low MCC with regard to some large proteins (e.g. Gelsolin, Kerato-epithilin, Lactoferrin) is the fact that only relative small regions of them have been studied and confirmed experimentally to be amyloidogenic. Therefore, there are too many false(?) positives for the rest of these proteins. We also see that most methods have problems with some prion proteins from fungi like Sup35, Ure2p and Het-s (Sup35 and Ure2p are Q/N-rich). But they seem to predict quite well the amyloidogenicity of the human Major prion protein. With the exception of Waltz, most methods predict different regions from the experimentally verified for Calcitonin (a 32-amino acid peptide hormone). They also seem to perform poorly for bacterial Cold Shock Protein from Bacillus subtilis, a small, completely amyloidogenic, protein (They predict only a small segment as amyloidogenic and therefore, there are many false negatives).
(PDF)
