# Supplementary material for: A Consensus Method for the Prediction of ‘Aggregation-Prone’ Peptides in Globular Proteins
Source: PLoS One. 2013 Jan 10;8(1):e54175. doi: 10.1371/journal.pone.0054175 (PMC3542318; doi:10.1371/journal.pone.0054175)
Supplement: Table S1 — Prediction of amyloidogenic regions or “aggregation-prone” stretches, for 33 amyloidogenic proteins by AMYLPRED and AMYLPRED2, for comparison. Superscripts after each protein name (first column) refer to the relevant literature used (given at the bottom of the Table) to obtain experimental information. The residue numbering for the sequence features (first column) refers to the respective Uniprot entries. The sequences of the mature proteins are given in the second column. Experimentally verified amyloid forming regions/“aggregation-prone” stretches are shown in bold. The residue numbering for the experimental and predicted regions (remaining columns) refers to the mature protein only. Bold font highlights hits that are in agreement with experimental data. Surface accessibility for these peptides was calculated in Å2, using DSSP, with a probe radius of 1.4 Å (which approximates the radius of a water molecule). One asterisk (*) denotes peptides on the surface of the relevant proteins using a per-residue cut-off of 20 Å2 (corresponding to ∼2 water molecules per residue). A double asterisk (**) denotes semi-surface peptides (with a per-residue value between 10–20 Å2). (PDF) [file pone.0054175.s001.pdf]

**Table S1**

| Protein | | Experimental Regions | AMYLPRED Hits | AMYLPRED2 Hits |
|---|---|---|---|---|
| | **Sequence** | | | |
| **Prolactin [1]** | LPICPG**GAARCQVTLRDLFDRAVVLSHYIHNLSS**EMFSEFDK**RYTHGRGFITKA** | 7-21*, 20-34* | **11-16*, 18-31*** | **11-16*, 20-32*** |
| **P01236 [29-227]** | **INS**CHTSSLATPEDKEQAQQMNQKDFLSLIVSILRSWNEPLYHLVTEVRGMQEA | | | |
| Signal Peptide | PEAILSKAVEIEEQTKRLLEGMELIVSQVHPETKENEIYPVWSGLPSLQMADEES | 43-57* | | **51-55*** |
| 1-28 | RLSAYYNLLHCLRRDSHKIDNYLKLLKCRIIHNNNC | | | |
| PDB code: 1RW5 | | | 80-89** | 80-89** |
| | | | 95-100* | 95-101* |
| | | | | 114-117* |
| | | | 130-136* | 129-137* |
| | | | | |
| | | | 167-176* | 167-173** |
| | | | 186-195* | 187-195* |

| | | | | |
|---|---|---|---|---|
| **Calcitonin [2,19]** | CGNLSTCMLGTYTQ**DFNKFH**TFPQTAIGVGAP | | 6-11 | 6-10 |
| **P01258 [85-116]** | | 15-19, 15-20 | | |
| Signal Peptide | | | | |
| 1-25 | | | | |
| Propeptide | | | | |
| 26-82 | | | | |
| Peptide (Katacalcin) | | | | |
| 121-141 | | | | |
| | | | | |

| Protein | | Experimental Regions | AMYLPRED Hits | AMYLPRED2 Hits |
|---|---|---|---|---|
| | **Sequence** | | | |
| **Apolipoprotein A-I [3]** | **DEPPQSPWDRVKDLATVYVDVLKDSGRDYVSQFEGSALGKQLNLKLLDNWD** | 1-93* | **14-22*** | **14-22*** |
| **P02647 [25-267]** | **SVTSTFSKLREQLGPVTQEFWDNLEKETEGLRQEMSKDLEEV**KAKVQPYLDD | | | |
| Signal Peptide | FQKKWQEEMELYRQKVEPLRAELQEGARQKLHELQEKLSPLGEEMRDRARAH | | **53-58*** | **53-57*** |
| 1-18 | VDALRTHLAPYSDELRQRLAARLEALKENGGARLAEYHAKATEHLSTLSEKAKP | | **69-72*** | |
| Propeptide | ALEDLRQGLLPVLESFKVSFLSALEEYTKKLNTQ | | | |
| 19-24 | | | | |
| **PDB code: 2A01** | | | | |
| | | | 227-232* | 224-232* |

| | | | | |
|---|---|---|---|---|
| **Casein (Bovine) [4]** | KNTMEHVSSSEESIISQETYKQEKNMAINPSKENLCSTFCKEVVRNANEEEYSI | | | |
| **P02663 [16-222]** | GSSSEESAEVATEEVKITVDDKHYQK**ALNEINQFYQKFPQYLQYLYQGPIVLNP** | | 35-40 | 35-39 |
| Signal Peptide | **WDQVKRNAVPITPTLNR**EQLSTSEENSKKTVDMESTEVFTKKTKLTEEEKNRL | | | |
| 1-15 | NFLKKISQRYQKFALPQYLKTVYQHQKAMKPWIQPKTKVIPYVRYL | 81-125 | **86-90, 95-100** | **86-88, 94-101** |
| | | | | |
| | | | | |
| | | | | 180-183 |
| | | | | 199-204 |
| | | | | |

| | | | | |
|---|---|---|---|---|
| **Serum Amyloid A [5-7]** | R**SFFSFLGEAFD**GARDMWRAYSDMREANYIGSDKYFHARGNYDAAKRGPGG | 1-12 | **2-8** | **2-9** |
| **P02735 [19-122]** | VWAAEAISDARENIQRFFGHGAEDSLADQAANEWGRSGKDPNHFRPAGLPEK | | | |
| Signal Peptide | Y | | | 53-55 |
| 1-18 | | | 68-70 | |

| | | | | |
|---|---|---|---|---|
| **Transthyretin [8-11]** | GPTGTGESK**CPLMVKVLDAV**RGSPAINVAVHVFRKAADDTWEPFASGKTSES | 10-20* | **11-17*** | **12-17*** |
| **P02766 [21-147]** | GELHGLTTEEEFVEGIYKVEIDTKSYWKALGISPFHEHAEVVFTANDSGPRR**YTI** | | 26-34* | 25-34* |
| Signal Peptide | **AALLSPYS**YSTTAVVTNPKE | | | |
| 1-20 | | | | |
| **PDB code: 1BMZ** | | | | |
| | | | 92-96** | 92-96** |
| | | 105-115* | **105-111*** | **105-112*, 114-115*** |
| | | | 118-122* | 117-123* |

| Protein | | Experimental Regions | AMYLPRED Hits | AMYLPRED2 Hits |
|---|---|---|---|---|
| | Sequence | | | |
| **Lactoferrin** [12] | GRRRSVQWCAVSQPEATKCFQWQRNMRKVRGPPVSCIKRDSPIQCIQAIAEN | | 6-10** | |
| **P02788 [20-710]** | RADAVTLDGGFIYEAGLAPYKLRPVAAEVYGTERQPRTHYYAVAVVKKGGSFQ | | 19-22* | 19-22* |
| Signal Peptide | LNELQGLKSCHTGLRRTAGWNVPIGTLRPFLNWTGPPEPIEAAVARFFSASCV | | 44-48* | 45-47* |
| 1-19 | PGADKGQFPNLCRLCAGTGENKCAFSSQEPYFSYSGAFKCLRDGAGDVAFIRE | | | 61-64*, 66 |
| **PDB code: 1CB6** | STVFEDLSDEAERDEYELLCPDNTRKPVDKFKDCHLARVPSHAVVARSVNGKE | | 91-98 | 92-99** |
| | DAIWNLLRQAQEKFGKDKSPKFQLFGSPSGQKDLLFKDSAIGFSRVPPRIDSGL | | | |
| | YLGSGYFTAIQNLRKSEEEVAARRARVVWCAVGEQELRKCNQWSGLSEGSVT | | 150-155* | 152-155** |
| | CSSASTTEDCIALVLKGEADAMSLDGGYVYTAGKCGLVPVLAENYKSQQSSDP | | | |
| | DPNCVDRPVEGYLAVAVVRRSDTSLTWNSVKGKKSCHTAVDRTAGWNIPMGL | | | 209-211* |
| | LFNQTGSCKFDEYFSQSCAPGSDPRSNLCALCIGDEQGENKCVPNSNERYYG | | | |
| | YTGAFRCLAE**NAGDVAFV**KDVTVLQNTDGNNNEAWAKDLKLADFALLCLDGK | | 254-258 | 254-257 |
| | RKPVTEARSCHLAMAPNHAVVSRMDKVERLKQVLLHQQAKFGRNGSDCPDKF | | 266-271* | 267-271* |
| | CLFQSETKNLLFNDNTECLARLHGKTTYEKYLGPQYVAGITNLKKCSTSPLLEA | | 286-290* | 286-290* |
| | CEFLRK | | | 301-302*, 305 |
| | | | | 322-329* |
| | | | 343-351** | 343-351** |
| | | | | |
| | | | 381-385** | 381-385** |
| | | | 406-410** | 406-408**, 410 |
| | | | 434-442** | 434-441 |
| | | | 473-478* | 473-478* |
| | | | 504-508* | 504-508* |
| | | | 532-534** | |
| | | 538-545* | **542-551** | **542-553** |
| | | | 569-576* | 569-576* |
| | | | 589-593* | |
| | | | 610-615* | 611-614* |
| | | | 630-635* | 630-635* |
| | | | 640-644** | |
| | | | | 667-672* |

| | | | | |
|---|---|---|---|---|
| **Major Prion Protein** [13-19] | KKRPKPGGWNTGGSRYPGQGSPGGNRYPPQGGGGWGQPHGGGWGQPHG | 84-104, 91-105 | | **99-104** |
| **P04156 [23-253]** | GGWGQPHGGGWGQPHGGGWGQGGGTHSQWNKPSKP **KTNMKHMAGAAA** | 105-125*, 116-122* | **115-119*** | **115-120*** |
| Signal Peptide | **AGAVVGGLGGYMLGSAMSRPIIHFGSDYED**RYYRENMHRYPNQVYYRPMDE | 148-153* | | |
| 1-22 | Y**SNQNNFVHDCVNITIKQHTVTTTT**KGENFTET**DVKMMERVVEQMCITQY**ERE | 154-163*, 156-171* | **152-162*** | **156-163*, 165-170*** |
| Propeptide | SQAYYQRGS**SMVLFSSPPVILLISFLIFLIVG** | 180-196* | **189-195*** | **187-195*** |
| 231-253 | | 209-231 | **209-214** | **208-215** |
| **PDB code: 1QLX** | | | **218-230** | **218-231** |

| | | | | |
|---|---|---|---|---|
| **Semenogelin I** [20] | **QKGGSKGRLPSEFSQFPHGQKGQHYSGQKGKQQTESKGSFSIQYTYHVDAN** | 1-142 | **40-48** | **40-48** |
| **P04279 [24-462]** | **DHDQSRKSQQYDLNALHKTTKSQRHLGGSQQLLHNKQEGRDHDKSKGHFH** | | **100-106** | **100-105** |
| Signal Peptide | **RVVIHHKGGKAHRGTQNPSQDQGNSPSGKGISSQYSNTEER**LWVHGLSKEQ | | | |
| 1-23 | TSVSGAQKGRKQGGSQSSYVLQTEELVANKQQRETKNSHQNKGHYQNVVEV | | 142-146 | |
| | REEHSSKVQTSLCPAHQDKLQHGSKDIFSTQDELLVYNKNQHQTKNLNQDQQ | | | 199-199 |
| | HGRKANKISYQSSSTEERRLHYGENGVQKDVSQSSIYSQTEEKAQGKSQKQITI | | | 237-241 |
| | PSQEQEHSQKANKISYQSSSTEERRLHYGENGVQKDVSQRSIYSQTEKLVAGK | | | |
| | SQIQAPNPKQEPWHGENAKGESGQSTNREQDLLSHEQKGRHQHGSHGGLDI | | 412-417 | 411-417 |

| Protein | | Experimental Regions | AMYLPRED Hits | AMYLPRED2 Hits |
|---|---|---|---|---|
| | Sequence | | | |
| **Beta-amyloid protein 42** [21-25,19] | DAEFRHDSGY**EVHHQKLVFFAEDVGSNKGAIIGLMVGGVVIA** | 11-25* | **16-21*** | **15-22*** |
| **P05067 [672-713]** | | 25-35* | | |
| Signal Peptide | | 30-40, 37-42* | **29-41*** | **29-41*** |
| 1-17 | | | | |
| **PDB code: 1IYT** | | | | |

| | | | | |
|---|---|---|---|---|
| **Gelsolin** [26-28] | ATASRGASQAGAPQGRVPEARPNSMVVEHPEFLKAGKEPGLQIWRVEKFDLV | | 42-46 | 44-46 |
| **P06396 [28-782]** | PVPTNLYGDFFTGDAYVILKTVQLRNGNLQYDLHYWLGNECSQDESGAAAIFT | | | |
| Signal Peptide | VQLDDYLNGRAVQHREVQGFESATFLGYFKSGLKYKKGGVASGFKHVVPNEV | | | 62-63 |
| 1-27 | VVQRLFQVKGRRVVR**ATEVPVSWESFNNGDCFILDLGNNIHQWCGSNSNRYE** | | 67-72 | 66-74 |
| **PDB code: 3FFN** | **RLKATQVSKGIRDNERSGRAR**VHVSEEGTEPEAMLQVLGPKPALPAGTEDTA | | 83-90 | |
| | KEDAANRKLAKLYKVSNGAGTMSVSLVADENPFAQGALKSEDCFILDHGKDGK | | 100-106* | 100-108* |
| | IFVWKGKQANTEERKAALKTASDFITKMDYPKQTQVSVLPEGGETPLFKQFFKN | | 128-134* | 128-134* |
| | WRDPDQTDGLGLSYLSSHIANVERVPFDAATLHTSTAMAAQHGMDDDGTGQK | | 157-163* | 157-163* |
| | QIWRIEGSNKVPVDPATYGQFYGGDSYIILYNYRHGGRQGQIIYNWQGAQSTQ | 173-230* | **186-192** | **186-191** |
| | DEVAASAILTAQLDEELGGTPVQSRVVQGKEPAHLMSLFGGKPMIIYKGGTSRE | | | |
| | GGQTAPASTRLFQVRANSAGATRAVEVLPKAGALNSNDAFVLKTPSAAYLWV | | 243-247** | |
| | GTGASEAEKTGAQELLRVLRAQPVQVAEGSEPDGFWEALGGKAAYRTSPRLK | | | |
| | DKKMDAHPPRLFACSNKIGRFVIEEVPGELMQEDLATDDVMLLDTWDQVFVW | | | |
| | VGKDSQEEEKTEALTSAKRYIETDPANRDRRTPITVVKQGFEPPSFVGWFLGW | | 303-308 | 303-308 |
| | DDDYWSVDPLDRAMAELAA | | | |
| | | | 361-366* | |
| | | | | 379-385, 388 |
| | | | | |
| | | | 445-453 | 445-453 |
| | | | 461-466 | 460-466 |
| | | | 480-484* | 479-484* |
| | | | 507-513 | 508-513 |
| | | | | 538-541 |
| | | | | |
| | | | | |
| | | | 574-580 | 575-581* |
| | | | 594-599* | 594-597* |
| | | | | |
| | | | 641-645 | 644-645 |
| | | | 652-654* | 650-655* |
| | | | 671-676 | |
| | | | 679-684 | 675-684** |
| | | | | |
| | | | 729-735* | 729-734* |

| Protein | | Experimental Regions | AMYLPRED Hits | AMYLPRED2 Hits |
|---|---|---|---|---|
| | **Sequence** | | | |
| **Tau** [29,30,19] | AEPRQEFEVMEDHAGTYGLGDRKDQGGYTMHQDQEGDTDAGLKESPLQTPT | | | |
| **P10636 [2-758]** | EDGSEEPGSETSDAKSTPTAEDVTAPLVDEGAPGKQAAAQPHTEIPEGTTAEE | | | |
| Initiator methionine removed | AGIGDTPSLEDEAAGHVTQEPESGKVVQEGFLREPGPPGLSHQLMSGMPGAP | | | |
| | LLPEGPREATRQPSGTGPEDTEGGRHAPELLKHQLLGDLHQEGPPLKGAGGK | | 298-305 | 299-307 |
| | ERPGSKEEVDEDRDVDESSPQDSPPSKASPAQDGRPPQTAAREATSIPGFPA | | | |
| | EGAIPLPVDFLSKVSTEIPASEPDGPSVGRAKGQDAPLEFTFHVEITPNVQKEQ | | | |
| | AHSEEHLGRAAFPGAPGEGPEARGPSLGEDTKEADLPEPSEKQPAAAPRGKP | 589-600 | **594-595** | **592-595** |
| | VSRVPQLKARMVSKSKDGTGSDDKKAKTSTRSSAKTLKNRPCLSPKLPTPGSS | | | |
| | DPLIQPSSPAVCPEPPSSPKHVSSVTSRTGSSGAKEMKLKGADGKTKIATPRG | 622-627 | **623-627** | **620-627** |
| | AAPPGQKGQANATRIPAKTPPAPKTPPSSGEPPKSGDRSGYSSPGSPGTPGS | | | |
| | RSRTPSLPTPPTREPKKVAVVRTPPKSPSSAKSRLQTAPVPMPDLKNVKSKIGS | | | 709 |
| | TENLKHQPGG**GKVQIINKKLDL**SNVQSKCGSKDNIKHVPGGGS**VQIVYK**PVDL | | | |
| | SKVTSKCGSLGNIHHKPGGGQVEVKSEKLDFKDRVQSKIGSLDNITHVPGGGN | | | |

| | | | | |
|---|---|---|---|---|
| **IAPP (Amylin)** [31-37,19] | KCNTATC**ATQRLANFLVHSSNNFGAILSSTNVGSNTY** | | 8-9* | |
| **P10997 [34-70]** | | 8-20*, 14-20* | **13-20*** | **13-21*** |
| Signal Peptide | | 20-29*, 21-30* | **23-24*** | **23-28*** |
| 1-22 | | 30-37* | | |
| Propeptide | | | | |
| 23-31 | | | | |
| Propeptide | | | | |
| 74-89 | | | | |
| **PDB code: 2L86** | | | | |

| | | | | |
|---|---|---|---|---|
| **Lung Surfactant** [38-40] | FGIPCCPV**HLKRLLIVVVVVVLIVVVIVGALLMG**L | 9-34 | 3-8, **10-33** | **12-33** |
| **Protein C** | | | | |
| **P11686 [24-58]** | | | | |
| Propeptide | | | | |
| 1-23 | | | | |
| Propeptide | | | | |
| 59-197 | | | | |

| Protein | | Experimental Regions | AMYLPRED Hits | AMYLPRED2 Hits |
|---|---|---|---|---|
| | Sequence | | | |
| **alpha-Synuclein** [41-49] | MDVFMKGLSKAKEGVVAAAEKTKQGVAEAAGKTK**EGVLYVGSKT**KEGV**VHGV** | | | |
| **P37840 [1-140]** | **ATVAEKTKEQVTNVGGAVVTGVTAVAQKTV**EGA**GSIAAATGFV**KKDQLGKNE | | 15-19* | 15-18* |
| | EGAPQEGILEDMPVDPDNEAYEMPSEEGYQDYEPEA | 35-95* (35-44* | **36-41*** | **36-41*** |
| **PDB code: 1XQ8** | | 49-59* | **52-53*** | **48-55*** |
| | | 60-68* | | |
| | | 69-82* | **69-78*** | **66-79*** |
| | | 86-95*) | | **87-94*** |
| | | | | |
| **Lysozyme C** [18,19] | KVFE**RCELARTLKR**LGMDGYRGIS**LANWMCLAKW**ESGYNTRATNYNAGDRS | 5-14* | | |
| **P61626 [19-148]** | TDYG**IFQINS**RYWCNDGKTPGAVNACHLSCSALLQDNIADAVACAKRVVRDPQ | 25-34* | 25-33** | 25-33** |
| Signal Peptide | GIRAWVAWRNRCQNRDVRQYVQGCGV | 56-61 | **55-59, 61-65*** | **55-62** |
| | | | 76-80* | |
| 1-18 | | | 82-84* | |
| **PDB code: 1LZ1** | | | 107-114* | 107-112* |
| | | | | |
| **beta2-Microglobulin** [50-57,19] | IQRTPKIQVYSRHPAENGK**SNFLNCYVSGFHPSDIEVDLLK**NGERIEKVEHSDL | 21-31* | **21-29**** | **21-30**** |
| **P61769 [21-119]** | SFSK**DWSFYLLYYTEFTPTEKDEYACRVNHVTLSQ**PKIVKWDRDM | 33-41* | | |
| Signal Peptide | | 59-71* | **60-69*** | **60-70*** |
| 1-20 | | 83-89* | **79-83*** | **80-87*** |
| **PDB code: 2YXF** | | 91-96 | | |
| | | | | |
| **Medin** [58-60] | RLDKQGNFNAWVAGSYGNDQWLQVDLGSSKE**VTGIITQGARNFGSVQFVA** | | 9-13 | 9-13 |
| **Q08431 [268-317]** | | 32-41 | **32-37** | **32-38** |
| Signal Peptide | | 42-50 | | **43-49** |
| 1-23 | | | | |
| | | | | |
| **proBNP** [61] | HPLGSPGSASDLETSGLQEQRNHLQGKLSELQVEQTSLEPLQESPRPTGVWK | | | |
| **P16860 [27-134]** | SREVATEGIRGHR**KMVLYTL**RAPRSPKMVQGSGCFGRKMDRISSSSGLGCKV | 66-72 | **66-72** | **66-72** |
| Signal Peptide | LRRH | | | |
| 1-26 | | | 102-105 | 102 |

| Protein | | Experimental Regions | AMYLPRED Hits | AMYLPRED2 Hits |
|---|---|---|---|---|
| | **Sequence** | | | |
| **Apolipoprotein C-II [62]** | TQQPQQDEMPSPTFLTQVKESLSSYWESAKTAAQNLYEKTYLPAVDEKLRDLY | | | 16-17* |
| **P02655 [23-101]** | SKSTAA**MSTYTGIFTDQ**VLSVLKGEE | | | |
| Signal Peptide | | 60-70* | **62-68*** | **62-75*** |
| 1-22 | | | | |
| **PDB code: 1O8T** | | | 71-75* | |
| | | | | |
| **ODAM [63]** | APLIPQRLMSASNSNELLLNLNNGQLLPLQLQGPLNSWIPPFSGILQQQQAQI | | 17-18 | |
| **A1E959 [16-279]** | PGLSQFSLSALDQFAGLLPNQIPLTGEASFAQGAQAGQVDPLQLQTPPQTQPG | | 60-63 | 60-62, 64, 68-69 |
| Signal Peptide | PSHV**MPYVFSFKMPQEQGQMFQYYPVYMVLPWEQPQQTVPRSPQQTRQQQ** | 112-157 | **112-118, 126-127, 130-139** | **113-118, 130-139** |
| 1-15 | YEEQIPFYAQFGYIPQLAEPAISGGQQQLAFDPQLGTAPEIAVMSTGEEIPYLQK | | 162-169 | 164-169 |
| | EAINFRHDSAGVFMPSTSPKPSTTNVFTSAVDQTITPELPEEKDKTDSLREP | | 199-200 | 199-200 |
| | | | 238-241 | 236-245 |
| | | | | |
| **Cystatin C [64,65]** | SSPGKPPRLVGGPMDASVEEEGVRRALDFAVGEYNKASNDMYHSRALQVVRA | | | |
| **P01034 [27-146]** | RKQIVAGVNYFLDVELGRTTCTKTQPNLDNCPFHDQPHLKRKAFC**SFQIYA**VP | | 47-51* | |
| Signal Peptide | WQGTMTLSKSTCQDA | | 56-65* | 56-66* |
| 1-26 | | 98-103* | **95-105*** | **96-105*** |
| **PDB code: 1G96** | | | | |
| | | | | |
| **Insulin [66,67,55,19]** | FVNQHLCGSH**LVEALYL**VCGERGFFYTPKT / GIVEQCCTSICS**LYQLEN**YCN | ***Chain B*** | ***Chain B*** | ***Chain B*** |
| **P01308 [25-54 / 90-110]** | | | 5-6* | |
| Signal Peptide | | 11-17** | **14-19**** | **11-19**** |
| 1-24 | | | 23-27* | 23-27* |
| Propeptide | | | | |
| 57-87 | | ***Chain A*** | ***Chain A*** | ***Chain A*** |
| **PDB code: 1ZNJ** | | 13-18* | 10-16* | 8, **10-16*** |

| Protein | | Experimental Regions | AMYLPRED Hits | AMYLPRED2 Hits |
|---|---|---|---|---|
| | **Sequence** | | | |
| **Beta-lactoglobulin [68]** | LIVTQTMKGL**DIQKVAGTWY**SLAMAASDISLLDAQSAPLRVYVEELKPTPEGDL | | 1-5 | 1-6 |
| **P02754 [17-178]** | EILLQKWENGECAQKKIIAEKTKIPAVFKIDALNENKVLVLDTDYK**KYLLFCMENS** | 11–20* | **17-24**** | **17-24**** |
| Signal Peptide | AEPEQ**SLACQCLVRTP**EVDDEALEKFDKALKALPM**HIRLSFN**PTQLEEQCHI | | 39-44**, 54-59** | 29-32*, 41-42, 55-58** |
| 1-16 | | | 93-97* | 80-81**, 83-84*, 92-96* |
| | | 101–110* | **100-107*** | **101-108*** |
| | | 116-126** | **117-124**** | **120-124**** |
| **PDB code: 1BEB** | | 146-152* | **145-151**** | **145-152*** |

| | | | | |
|---|---|---|---|---|
| **Acylphosphatase-2 [69,70,71]** | STAQSLKSVDYEVFG**RVQGVCFRMYTEDEAR**KIGVVGWVKNTSKGTVTGQVQ | | | |
| **P14621 [2-99]** | GPEDKVNSMKSWLSKVGSPSSRIDRTNFSNEKTI**SKLEYSNFSIRY** | 16-31 | **19-25** | **17-24** |
| Initiator methionine removed | | | 34-39 | 33-39 |
| | | | | |
| | | 87-98 | **92-95** | **91-95** |

| | | | | |
|---|---|---|---|---|
| **Amphoterin (Rat) [72]** | GKGDPKKPRGK**MSSYAFFVQTCREEHK**KKHPDASVNFSEFSKKCSERWKTM | | | |
| **P63159 [2-215]** | SAKEKGKFEDMAKADKARYEREMKTYIPPKGETKKKFKDPNAPKRPPSAFFLF | 12-27* | **14-21*** | **14-21*** |
| Initiator methionine removed | CSEYRPKIKGEHPGLSIGDVAKKLGEMWNNTAADDKQPYEKKAAKLKEKYEKD | | | 34-38* |
| | IAAYRAKGKPDAAKKGVVKAEKSKKKKEEEDDEEDEEDEEEEEEEEDEDEEED | | 99-106 | 99-106 |
| **PDB code: 1CKT** | DDDE | | | |

| | | | | |
|---|---|---|---|---|
| **Cold shock protein CspB [73,74]** | **MLEGKVKWFNSEKGFGFIEVEGQDDVFVHFSAIQGEGFKTLEEGQAVSFEIVE** | 1-22*, 1-35* | **26-32*** | **26-32*** |
| (*Bacillus subtilis*) | **GNRGPQAANVTKEA** | 36-67* | **47-52*** | **47-52*** |
| **P32081[1-67]** | | | | |
| | | | | |
| **PDB code: 3PF4** | | | | |

| Protein | | Experimental Regions | AMYLPRED Hits | AMYLPRED2 Hits |
|---|---|---|---|---|
| | **Sequence** | | | |
| **Kerato-epithelin** [75] | GPAKSPYQLVLQHSRLRGRQHGPNVCAVQKVIGTNRKYFTNCKQWYQRKICG | | 8-12 | 8-12 |
| **Q15582 [24-683]** | KSTVISYECCPGYEKVPGEKGCPAALPLSNLYETLGVVGSTTTQLYTDRTEKLR | | 27-31, 54-61 | 27-31, 54-61 |
| Signal Peptide | PEMEGPGSFTIFAPSNEAWASLPAEVLDSLVSNVNIELLNALRYHMVGRRVLTD | | | 80-94 |
| 1-23 | ELKHGMTLTSMYQNSNIQIHHYPNGIVTVNCARLLKADHHATNGVVHLIDKVISTI | | 113-119 | 113-119 |
| | TNNIQQIIEIEDTFETLRAAVAASGLNTMLEGNGQYTLLAPTNEAFEKIPSETLNRI | | 142-143, 148-155 | 138-150, 153 |
| | LGDPEALRDLLNNHILKSAMCAEAIVAGLSVETLEGTTLEVGCSGDMLTINGKAII | | 177-181, 191-192 | 176-179, 185-192 |
| **PDB code: 1X3B** | SNKDILATNGVIHYIDELLIPDSAKTLFELAAESDVSTAIDLFRQAGLGNHLSGSE | | 206-210, 212-218, 221-225 | 204-225 |
| | RLTLLAPLNSVFKDGTPPIDAHTRNLLRNHIIKDQLASKYLYHGQTLETLGGKKL | | 252-256 | 254-256 |
| | RVFVYRNSLCIENSCIAAHDKRGRYGTLFTMDRVLTPPMGTVMDVLKGDNR**FS** | | 297-300 | 297-303 |
| | **MLVAAIQSAGLTETLN**REGVYTVFAPTNEAFRALPPRERSRLLGDAKELANILK | | 340-345 | 323-327, 336-348 |
| | YHIGDEILVSGGIGALVRLKSLQGDKLEVSLKNNVVSVNKEPVAEPDIMATNGVV | | 371-372, 387-392 | 371, 387-391 |
| | HVITNVLQPPANRPQERGDELADSALEIFKQASAFSRASQRSVRLAPVYQKLLE | | 425-428 | |
| | RMKH | | 441-447, 466-472 | 441-450, 467-472 |
| | | 492-502*, 492-509* | **492-499*** | **492-500*** |
| | | | 512-518* | 512-518*, |
| | | | 545-547* | 544-547*, 549-550 |
| | | | 561-565* | 555-565* |
| | | | | 581-584* |
| | | | 602-609*, 628-631 | 600-609**, 628-631 |

| | | | | |
|---|---|---|---|---|
| **Myoglobin (Horse)** [76,77] | **GLSDGEWQQVLNVWGKVEADIAGHGQEVL**IRLFTGHPETLEKFDKFKHLKTEA | 1-29* | **10-11*, 28-34*** | **10-15*, 27-34*** |
| **P68082[2-154]** | EMKASEDLKKHGTVVLTALGGILKKKGHHEAELKPLAQSHATKHKIP**IKYLEFIS** | | | |
| Initiator methionine removed | **DAIIHVLHSK**HPGDFGADAQGAMTKALELFRNDIAAKYKELGFQG | | 66-73* | 66-75* |
| | | 101-118* | **102-108*,110-116*** | **101-116*** |
| **PDB code: 1WLA** | | | | |

| | | | | |
|---|---|---|---|---|
| **RepA (*P. syringae*)** [78] | NEKR**LVLCAASLI**DSRKPLPKDGYLTIRADTFAEVFGIDVKHAYAALDDAATKLF | 5-13* | **4-13*** | **5-12*** |
| **Q52546 [23-231]** | NRDIRRYVKGKVVERMRWVFHVKYREGQGCVELGFSPTIIPHLTMLHKEFTSY | | 53-54*, 71-78** | 35-36*, 71-78** |
| Signal peptide | QLKQIGSLSSFYAVRLYELMSQFIKLKQRECTLAQLREMFDLGDKYQDVKDMR | | 95-102* | 93-98* |
| 1-22 | KRVLYPALEEVNKNTDLTVAVEPRRQGRRIIGFSFTIAKNDQLALSLE | | | 109-110* |
| | | | 116-127 | 116-127 |
| | | | 130-133 | 129-133 |
| | | | 162-167, 178-181 | |
| **PDB code: 1HKQ** | | | 191-199 | 191-199 |

| Protein | Sequence | Experimental Regions | AMYLPRED Hits | AMYLPRED2 Hits |
|---|---|---|---|---|
| **Sup35 (*S. cerevisiae* )** [79,80,19] | MSDSNQ**GNNQQNY**QQYSQNGNQQQGNNRYQGYQAYNAQAQPAGGYYQNY | 7-13 | | |
| **P05453 [1-685]** | QGYSGYQQGGYQQYNPDAGYQQQYNPQGGYQQYNPQGGYQQQFNPQGG | | 102 - 106 | |
| | RGNYKNFNYNNNLQGYQAGFQPQSQGMSLNDFQKQQKQAAPKPKKTLKLVS | | 260 - 267 | 260-268 |
| | SSGIKLANATKKVGTKPAESDKKEEEKSAETKEPTKEPTKVEEPVKKEEKPVQT | | 278 - 284 | 278-285 |
| | EEKTEEKSELPKVEDLKISESTHNTNNANVTSADALIKEQEEEVDDEVVNDMFG | | 306 - 313 | 306-313 |
| | GKDHVSLIFMGHVDAGKSTMGGNLLYLTGSVDKRTIEKYEREAKDAGRQGWY | | 364 - 371 | 363-371, 398-407 |
| | LSWVMDTNKEERNDGKTIEVGKAYFETEKRRYTILDAPGHKMYVSEMIGGASQ | | 400 - 406 | |
| | ADVGVLVISARKGEYETGFERGGQTREHALLAKTQGVNKMVVVVNKMDDPTV | | 426 - 431, 442 - 446 | 426-436, 440-448 |
| | NWSKERYDQCVSNVSNFLRAIGYNIKTDVVFMPVSGYSGANLKDHVDPKECP | | 490 - 494 | |
| | WYTGPTLLEYLDTMNHVDRHINAPFMLPIAAKMKDLGTIVEGKIESGHIKKGQST | | 529 - 530 | 529-534 |
| | LLMPNKTAVEIQNIYNETENEVDMAMCGEQVKLRIKGVEEEDISPGFVLTSPKN | | 580 - 591 | 566-571 |
| | PIKSVTKFVAQIAIVELKSIIAAGFSCVMHVHTAIEEVHIVKLLHKLEKGTNRKSKK | | 593 - 607, 612 - 618 | 578-618 |
| | PPAFAKKGMKVIAVLETEAPVCVETYQDYPQLGRFTLRDQGTTIAIGKIVKIAE | | 640 - 646, 653 - 657 | 640-648, 654-654 |
| | | | | 672-684 |
| | | | | |
| **Ure2p (*S. cerevisiae* )** [81,82] | **MMNNNGNQVSNLSNALRQVNIGNRNSNTTTDQSNINFEFSTGVNNNNNNNSS** | 1-89, 10-39 | **35-39** | **35-39** |
| **P23202 [1-354]** | **SNNNNVQNNNSGRNGSQNNDNENNIKNTLEQHRQQQQ**AFSDMSHVEYSRIT | | 127-133, 136-146* | 126-147* |
| | KFFQEQPLEGYTLFSHRSAPNGFKVAIVLSELGFHYNTIFLDFNLGEHRAPEFVS | | 184-190 | 183-190 |
| | VNPNARVPALIDHGMDNLSIWESGAILLHLVNKYYKETGNPLLWSDDLADQSQI | | 212-219, 231-237* | 212-219, 231-236* |
| | NAWLFFQTSGHAPMIGQALHFRYFHSQKIASAVERYTDEVRRVYGVVEMALAE | | 255-263 | 255-261** |
| | RREALVMELDTENAAAYSAGTTPMSQSRFFDYPVWLVGDKLTIADLAFVPWNN | | 297-303* | 295-303* |
| | VVDRIGINIKIEFPEVYKWTKHMMRRPAVIKALRGE | | 311-317 | 306-314, 316-318* |
| **PDB code: 1G6W** | | | | 323-330* |
| | | | | |
| **Het-s (*P. anserina* )** [83,84] | MSEPFGIVAGALNVAGLFNNCVDCFEYVQLGRPFGRDYERCQLRLDIAKARLS | | 7-20, 22-28* | 7-29 |
| **Q03689 [1-289]** | RWGEAVKINDDPRFHSDAPTDKSVQLAKSIVEEILLLFESAQKTSKRYELVADQ | | 41-44 | |
| | QDLVVFEDKDMKPIGRALHRRLNDLVSRRQKQTSLAKKTAWALYDGKSLEKIV | | 85-92* | 83-93* |
| | DQVARFVDELEKAFPIEAVCHKLAEIEIEEVEDEASLTILKDAAGGIDAAMSDAAA | | 147-151 | |
| | Q**KIDAIVGRNSAKDIRTEERARVQLGNVVTAAALHGGIRISDQTTNSVETVVGK** | | 178-182* | |
| | **GESRVLIGNEYGGKGFWDN** | | 195-200* | 195-200* |
| | | 218-289* | **220-221*** | **219-224*, 242-250*** |
| **PDB code: 2WVN / 2KJ3** | | | **244-250*** | **266-267*, 277-280** |

## REFERENCES


1. Westermark P, Eriksson L, Engstrom U, Enestrom S, Sletten K (1997) Prolactin-derived amyloid in the aging pituitary gland. Am J Pathol 150: 67-73.
2. Reches M, Porat Y, Gazit E (2002) Amyloid fibril formation by pentapeptide and tetrapeptide fragments of human calcitonin. J Biol Chem 277: 35475-35480.
3. Andreola A, Bellotti V, Giorgetti S, Mangione P, Obici L, et al. (2003) Conformational switching and fibrillogenesis in the amyloidogenic fragment of apolipoprotein a-I. J Biol Chem 278: 2444-2451.
4. Niewold TA, Murphy CL, Hulskamp-Koch CA, Tooten PC, Gruys E (1999) Casein related amyloid, characterization of a new and unique amyloid protein isolated from bovine corpora amylacea. Amyloid 6: 244-249.
5. Westermark GT, Engstrom U, Westermark P (1992) The N-terminal segment of protein AA determines its fibrillogenic property. Biochem Biophys Res Commun 182: 27-33.
6. Patel H, Bramall J, Waters H, De Beer MC, Woo P (1996) Expression of recombinant human serum amyloid A in mammalian cells and demonstration of the region necessary for high-density lipoprotein binding and amyloid fibril formation by site-directed mutagenesis. Biochem J 318 ( Pt 3): 1041-1049.
7. Rubin N, Perugia E, Wolf SG, Klein E, Fridkin M, et al. (2010) Relation between serum amyloid A truncated peptides and their suprastructure chirality. J Am Chem Soc 132: 4242-4248.
8. Gustavsson A, Engstrom U, Westermark P (1991) Normal transthyretin and synthetic transthyretin fragments form amyloid-like fibrils in vitro. Biochem Biophys Res Commun 175: 1159-1164.
9. Jarvis JA, Craik DJ, Wilce MC (1993) X-ray diffraction studies of fibrils formed from peptide fragments of transthyretin. Biochem Biophys Res Commun 192: 991-998.
10. Chamberlain AK, MacPhee CE, Zurdo J, Morozova-Roche LA, Hill HA, et al. (2000) Ultrastructural organization of amyloid fibrils by atomic force microscopy. Biophys J 79: 3282-3293.
11. Jaroniec CP, MacPhee CE, Astrof NS, Dobson CM, Griffin RG (2002) Molecular conformation of a peptide fragment of transthyretin in an amyloid fibril. Proc Natl Acad Sci U S A 99: 16748-16753.
12. Nilsson MR, Dobson CM (2003) In vitro characterization of lactoferrin aggregation and amyloid formation. Biochemistry (Mosc) 42: 375-382.
13. Gasset M, Baldwin MA, Lloyd DH, Gabriel JM, Holtzman DM, et al. (1992) Predicted alpha-helical regions of the prion protein when synthesized as peptides form amyloid. Proc Natl Acad Sci U S A 89: 10940-10944.
14. Forloni G, Angeretti N, Chiesa R, Monzani E, Salmona M, et al. (1993) Neurotoxicity of a prion protein fragment. Nature 362: 543-546.
15. Thompson A, White AR, McLean C, Masters CL, Cappai R, et al. (2000) Amyloidogenicity and neurotoxicity of peptides corresponding to the helical regions of PrP(C). J Neurosci Res 62: 293-301.
16. Florio T, Paludi D, Villa V, Principe DR, Corsaro A, et al. (2003) Contribution of two conserved glycine residues to fibrillogenesis of the 106-126 prion protein fragment. Evidence that a soluble variant of the 106-126 peptide is neurotoxic. J Neurochem 85: 62-72.
17. Satheeshkumar KS, Jayakumar R (2003) Conformational polymorphism of the amyloidogenic peptide homologous to residues 113-127 of the prion protein. Biophys J 85: 473-483.
18. Fernandez-Escamilla AM, Rousseau F, Schymkowitz J, Serrano L (2004) Prediction of sequence-dependent and mutational effects on the aggregation of peptides and proteins. Nat Biotechnol 22: 1302-1306.
19. Sawaya MR, Sambashivan S, Nelson R, Ivanova MI, Sievers SA, et al. (2007) Atomic structures of amyloid cross-beta spines reveal varied steric zippers. Nature 447: 453-457.
20. Linke RP, Joswig R, Murphy CL, Wang S, Zhou H, et al. (2005) Senile seminal vesicle amyloid is derived from semenogelin I. J Lab Clin Med 145: 187-193.
21. Pike CJ, Walencewicz-Wasserman AJ, Kosmoski J, Cribbs DH, Glabe CG, et al. (1995) Structure-activity analyses of beta-amyloid peptides: contributions of the beta 25-35 region to aggregation and neurotoxicity. J Neurochem 64: 253-265.
22. Wood SJ, Wetzel R, Martin JD, Hurle MR (1995) Prolines and amyloidogenicity in fragments of the Alzheimer's peptide beta/A4. Biochemistry (Mosc) 34: 724-730.
23. Tjernberg LO, Callaway DJ, Tjernberg A, Hahne S, Lilliehook C, et al. (1999) A molecular model of Alzheimer amyloid beta-peptide fibril formation. J Biol Chem 274: 12619-12625.
24. Balbach JJ, Ishii Y, Antzutkin ON, Leapman RD, Rizzo NW, et al. (2000) Amyloid fibril formation by A beta 16-22, a seven-residue fragment of the Alzheimer's beta-amyloid peptide, and structural characterization by solid state NMR. Biochemistry (Mosc) 39: 13748-13759.
25. Petkova AT, Buntkowsky G, Dyda F, Leapman RD, Yau WM, et al. (2004) Solid state NMR reveals a pH-dependent antiparallel beta-sheet registry in fibrils formed by a beta-amyloid peptide. J Mol Biol 335: 247-260.
26. Maury CP, Nurmiaho-Lassila EL, Boysen G, Liljestrom M (2003) Fibrillogenesis in gelsolin-related familial amyloidosis. Amyloid 10 Suppl 1: 21-25.
27. Fadika GO, Baumann M (2002) Peptides corresponding to gelsolin derived amyloid of the finnish type (AGelFIN) adopt two distinct forms in solution of which only one can polymerize into amyloid fibrils and form complexes with apoE. Amyloid 9: 75-82.
28. Solomon JP, Yonemoto IT, Murray AN, Price JL, Powers ET, et al. (2009) The 8 and 5 kDa fragments of plasma gelsolin form amyloid fibrils by a nucleated polymerization mechanism, while the 68 kDa fragment is not amyloidogenic. Biochemistry (Mosc) 48: 11370-11380.
29. von Bergen M, Friedhoff P, Biernat J, Heberle J, Mandelkow EM, et al. (2000) Assembly of tau protein into Alzheimer paired helical filaments depends on a local sequence motif ((306)VQIVYK(311)) forming beta structure. Proc Natl Acad Sci U S A 97: 5129-5134.
30. von Bergen M, Barghorn S, Li L, Marx A, Biernat J, et al. (2001) Mutations of tau protein in frontotemporal dementia promote aggregation of paired helical filaments by enhancing local beta-structure. J Biol Chem 276: 48165-48174.
31. Westermark P, Engstrom U, Johnson KH, Westermark GT, Betsholtz C (1990) Islet amyloid polypeptide: pinpointing amino acid residues linked to amyloid fibril formation. Proc Natl Acad Sci U S A 87: 5036-5040.
32. Nilsson MR, Raleigh DP (1999) Analysis of amylin cleavage products provides new insights into the amyloidogenic region of human amylin. J Mol Biol 294: 1375-1385.
33. Tenidis K, Waldner M, Bernhagen J, Fischle W, Bergmann M, et al. (2000) Identification of a penta- and hexapeptide of islet amyloid polypeptide (IAPP) with amyloidogenic and cytotoxic properties. J Mol Biol 295: 1055-1071.
34. Goldsbury C, Goldie K, Pellaud J, Seelig J, Frey P, et al. (2000) Amyloid fibril formation from full-length and fragments of amylin. J Struct Biol 130: 352-362.
35. Azriel R, Gazit E (2001) Analysis of the minimal amyloid-forming fragment of the islet amyloid polypeptide. An experimental support for the key role of the phenylalanine residue in amyloid formation. J Biol Chem 276: 34156-34161.
36. Jaikaran ET, Higham CE, Serpell LC, Zurdo J, Gross M, et al. (2001) Identification of a novel human islet amyloid polypeptide beta-sheet domain and factors influencing fibrillogenesis. J Mol Biol 308: 515-525.
37. Mazor Y, Gilead S, Benhar I, Gazit E (2002) Identification and characterization of a novel molecular-recognition and self-assembly domain within the islet amyloid polypeptide. J Mol Biol 322: 1013-1024.
38. Szyperski T, Vandenbussche G, Curstedt T, Ruysschaert JM, Wuthrich K, et al. (1998) Pulmonary surfactant-associated polypeptide C in a mixed organic solvent transforms from a monomeric alpha-helical state into insoluble beta-sheet aggregates. Protein Sci 7: 2533-2540.
39. Gustafsson M, Thyberg J, Naslund J, Eliasson E, Johansson J (1999) Amyloid fibril formation by pulmonary surfactant protein C. FEBS Lett 464: 138-142.
40. Johansson J (2003) Molecular determinants for amyloid fibril formation: lessons from lung surfactant protein C. Swiss medical weekly 133: 275-282.
41. Han H, Weinreb PH, Lansbury PT, Jr. (1995) The core Alzheimer's peptide NAC forms amyloid fibrils which seed and are seeded by beta-amyloid: is NAC a common trigger or target in neurodegenerative disease? Chem Biol 2: 163-169.
42. Bodles AM, Guthrie DJ, Harriott P, Campbell P, Irvine GB (2000) Toxicity of non-abeta component of Alzheimer's disease amyloid, and N-terminal fragments thereof, correlates to formation of beta-sheet structure and fibrils. Eur J Biochem 267: 2186-2194.
43. Giasson BI, Murray IV, Trojanowski JQ, Lee VM (2001) A hydrophobic stretch of 12 amino acid residues in the middle of alpha-synuclein is essential for filament assembly. J Biol Chem 276: 2380-2386.
44. el-Agnaf OM, Irvine GB (2002) Aggregation and neurotoxicity of alpha-synuclein and related peptides. Biochem Soc Trans 30: 559-565.
45. Du HN, Tang L, Luo XY, Li HT, Hu J, et al. (2003) A peptide motif consisting of glycine, alanine, and valine is required for the fibrillization and cytotoxicity of human alpha-synuclein. Biochemistry (Mosc) 42: 8870-8878.

46. Bodles AM, Irvine GB (2004) Alpha-synuclein aggregation. Protein and peptide letters 11: 271-279.
47. Heise H, Hoyer W, Becker S, Andronesi OC, Riedel D, et al. (2005) Molecular-level secondary structure, polymorphism, and dynamics of full-length alpha-synuclein fibrils studied by solid-state NMR. Proc Natl Acad Sci U S A 102: 15871-15876.
48. Vilar M, Chou HT, Luhrs T, Maji SK, Riek-Loher D, et al. (2008) The fold of alpha-synuclein fibrils. Proc Natl Acad Sci U S A 105: 8637-8642.
49. Yagi H, Takeuchi H, Ogawa S, Ito N, Sakane I, et al. (2010) Isolation of short peptide fragments from alpha-synuclein fibril core identifies a residue important for fibril nucleation: a possible implication for diagnostic applications. Biochim Biophys Acta 1804: 2077-2087.
50. Kozhukh GV, Hagihara Y, Kawakami T, Hasegawa K, Naiki H, et al. (2002) Investigation of a peptide responsible for amyloid fibril formation of beta 2-microglobulin by achromobacter protease I. J Biol Chem 277: 1310-1315.
51. Jones S, Manning J, Kad NM, Radford SE (2003) Amyloid-forming peptides from beta2-microglobulin-Insights into the mechanism of fibril formation in vitro. J Mol Biol 325: 249-257.
52. Hasegawa K, Ohhashi Y, Yamaguchi I, Takahashi N, Tsutsumi S, et al. (2003) Amyloidogenic synthetic peptides of beta2-microglobulin--a role of the disulfide bond. Biochem Biophys Res Commun 304: 101-106.
53. Ivanova MI, Gingery M, Whitson LJ, Eisenberg D (2003) Role of the C-terminal 28 residues of beta2-microglobulin in amyloid fibril formation. Biochemistry (Mosc) 42: 13536-13540.
54. Ivanova MI, Sawaya MR, Gingery M, Attinger A, Eisenberg D (2004) An amyloid-forming segment of beta2-microglobulin suggests a molecular model for the fibril. Proc Natl Acad Sci U S A 101: 10584-10589.
55. Ivanova MI, Thompson MJ, Eisenberg D (2006) A systematic screen of beta(2)-microglobulin and insulin for amyloid-like segments. Proc Natl Acad Sci U S A 103: 4079-4082.
56. Platt GW, Routledge KE, Homans SW, Radford SE (2008) Fibril growth kinetics reveal a region of beta2-microglobulin important for nucleation and elongation of aggregation. J Mol Biol 378: 251-263.
57. Routledge KE, Tartaglia GG, Platt GW, Vendruscolo M, Radford SE (2009) Competition between intramolecular and intermolecular interactions in an amyloid-forming protein. J Mol Biol 389: 776-786.
58. Haggqvist B, Naslund J, Sletten K, Westermark GT, Mucchiano G, et al. (1999) Medin: an integral fragment of aortic smooth muscle cell-produced lactadherin forms the most common human amyloid. Proc Natl Acad Sci U S A 96: 8669-8674.
59. Reches M, Gazit E (2004) Amyloidogenic hexapeptide fragment of medin: homology to functional islet amyloid polypeptide fragments. Amyloid 11: 81-89.
60. Larsson A, Soderberg L, Westermark GT, Sletten K, Engstrom U, et al. (2007) Unwinding fibril formation of medin, the peptide of the most common form of human amyloid. Biochem Biophys Res Commun 361: 822-828.
61. Iconomidou VA, Pheida D, Hamodraka ES, Antony C, Hoenger A, et al. (2011) An amyloidogenic determinant in N-terminal pro-brain natriuretic peptide (NT-proBNP): Implications for cardiac amyloidoses. Biopolymers.
62. Wilson LM, Mok YF, Binger KJ, Griffin MD, Mertens HD, et al. (2007) A structural core within apolipoprotein C-II amyloid fibrils identified using hydrogen exchange and proteolysis. J Mol Biol 366: 1639-1651.
63. Murphy CL, Kestler DP, Foster JS, Wang S, Macy SD, et al. (2008) Odontogenic ameloblast-associated protein nature of the amyloid found in calcifying epithelial odontogenic tumors and unerupted tooth follicles. Amyloid 15: 89-95.
64. Ghiso J, Jensson O, Frangione B (1986) Amyloid fibrils in hereditary cerebral hemorrhage with amyloidosis of Icelandic type is a variant of gamma-trace basic protein (cystatin C). Proc Natl Acad Sci U S A 83: 2974-2978.
65. Pastor MT, Kummerer N, Schubert V, Esteras-Chopo A, Dotti CG, et al. (2008) Amyloid toxicity is independent of polypeptide sequence, length and chirality. J Mol Biol 375: 695-707.
66. Storkel S, Schneider HM, Muntefering H, Kashiwagi S (1983) Iatrogenic, insulin-dependent, local amyloidosis. Lab Invest 48: 108-111.
67. Ivanova MI, Sievers SA, Sawaya MR, Wall JS, Eisenberg D (2009) Molecular basis for insulin fibril assembly. Proc Natl Acad Sci U S A 106: 18990-18995.
68. Hamada D, Tanaka T, Tartaglia GG, Pawar A, Vendruscolo M, et al. (2009) Competition between folding, native-state dimerisation and amyloid aggregation in beta-lactoglobulin. J Mol Biol 386: 878-890.
69. Chiti F, Taddei N, Baroni F, Capanni C, Stefani M, et al. (2002) Kinetic partitioning of protein folding and aggregation. Nat Struct Biol 9: 137-143.
70. Chiti F, Calamai M, Taddei N, Stefani M, Ramponi G, et al. (2002) Studies of the aggregation of mutant proteins in vitro provide insights into the genetics of amyloid diseases. Proc Natl Acad Sci U S A 99 Suppl 4: 16419-16426.
71. Bemporad F, Taddei N, Stefani M, Chiti F (2006) Assessing the role of aromatic residues in the amyloid aggregation of human muscle acylphosphatase. Protein Sci 15: 862-870.
72. Kallijarvi J, Haltia M, Baumann MH (2001) Amphoterin includes a sequence motif which is homologous to the Alzheimer's beta-amyloid peptide (Abeta), forms amyloid fibrils in vitro, and binds avidly to Abeta. Biochemistry (Mosc) 40: 10032-10037.
73. Gross M, Wilkins DK, Pitkeathly MC, Chung EW, Higham C, et al. (1999) Formation of amyloid fibrils by peptides derived from the bacterial cold shock protein CspB. Protein Sci 8: 1350-1357.
74. Wilkins DK, Dobson CM, Gross M (2000) Biophysical studies of the development of amyloid fibrils from a peptide fragment of cold shock protein B. Eur J Biochem 267: 2609-2616.
75. Yuan C, Berscheit HL, Huang AJ (2007) Identification of an amyloidogenic region on keratoepithelin via synthetic peptides. FEBS Lett 581: 241-247.
76. Picotti P, De Franceschi G, Frare E, Spolaore B, Zambonin M, et al. (2007) Amyloid fibril formation and disaggregation of fragment 1-29 of apomyoglobin: insights into the effect of pH on protein fibrillogenesis. J Mol Biol 367: 1237-1245.
77. Fandrich M, Forge V, Buder K, Kittler M, Dobson CM, et al. (2003) Myoglobin forms amyloid fibrils by association of unfolded polypeptide segments. Proc Natl Acad Sci U S A 100: 15463-15468.
78. Giraldo R (2007) Defined DNA sequences promote the assembly of a bacterial protein into distinct amyloid nanostructures. Proc Natl Acad Sci U S A 104: 17388-17393.
79. Balbirnie M, Grothe R, Eisenberg DS (2001) An amyloid-forming peptide from the yeast prion Sup35 reveals a dehydrated beta-sheet structure for amyloid. Proc Natl Acad Sci U S A 98: 2375-2380.
80. Gsponer J, Haberthur U, Caflisch A (2003) The role of side-chain interactions in the early steps of aggregation: Molecular dynamics simulations of an amyloid-forming peptide from the yeast prion Sup35. Proc Natl Acad Sci U S A 100: 5154-5159.
81. Chan JC, Oyler NA, Yau WM, Tycko R (2005) Parallel beta-sheets and polar zippers in amyloid fibrils formed by residues 10-39 of the yeast prion protein Ure2p. Biochemistry (Mosc) 44: 10669-10680.
82. Baxa U, Wickner RB, Steven AC, Anderson DE, Marekov LN, et al. (2007) Characterization of beta-sheet structure in Ure2p1-89 yeast prion fibrils by solid-state nuclear magnetic resonance. Biochemistry (Mosc) 46: 13149-13162.
83. Balguerie A, Dos Reis S, Ritter C, Chaignepain S, Coulary-Salin B, et al. (2003) Domain organization and structure-function relationship of the HET-s prion protein of Podospora anserina. EMBO J 22: 2071-2081.
84. Van Melckebeke H, Wasmer C, Lange A, Ab E, Loquet A, et al. (2010) Atomic-resolution three-dimensional structure of HET-s(218-289) amyloid fibrils by solid-state NMR spectroscopy. J Am Chem Soc 132: 13765-13775
