# Supplementary material for: A Consensus Method for the Prediction of ‘Aggregation-Prone’ Peptides in Globular Proteins
Source: PLoS One. 2013 Jan 10;8(1):e54175. doi: 10.1371/journal.pone.0054175 (PMC3542318; doi:10.1371/journal.pone.0054175)
Supplement: Table S2 — MCC per protein per method. The main reason that the majority of methods has a low MCC with regard to some large proteins (e.g. Gelsolin, Kerato-epithilin, Lactoferrin) is the fact that only relative small regions of them have been studied and confirmed experimentally to be amyloidogenic. Therefore, there are too many false(?) positives for the rest of these proteins. We also see that most methods have problems with some prion proteins from fungi like Sup35, Ure2p and Het-s (Sup35 and Ure2p are Q/N-rich). But they seem to predict quite well the amyloidogenicity of the human Major prion protein. With the exception of Waltz, most methods predict different regions from the experimentally verified for Calcitonin (a 32-amino acid peptide hormone). They also seem to perform poorly for bacterial Cold Shock Protein from Bacillus subtilis, a small, completely amyloidogenic, protein (They predict only a small segment as amyloidogenic and therefore, there are many false negatives). (PDF) [file pone.0054175.s002.pdf]

Table S2

Protein	Aggrescan	Amyloid Mutants	Amyloidogenic pattern	Average Packing Density	Beta-strand contiguity	Hexapeptide Conf. Energy	NetCSSP	Pafig	SecStr	Tango	Waltz	AMYLPRED	AMYLPRED2
Acylphosphatase-2	0.35	-0.05	0.40	0.25	0.07	0.28	0.09	0.01	0.00	-0.15	0.29	0.37	0.39
Amphoterin (rat)	0.39	0.23	-0.08	0.48	0.69	0.52	0.29	-0.09	0.42	0.43	0.34	0.46	0.38
Amylin (IAPP)	0.31	0.02	0.21	0.21	0.14	-0.21	0.40	0.62	0.17	0.00	0.79	0.33	0.40
Apolipoprotein A-I	0.02	0.19	0.16	0.11	0.26	0.10	0.27	0.61	0.05	0.26	0.24	0.26	0.15
Apolipoprotein C-II	0.59	0.14	0.34	0.71	0.74	0.51	0.39	0.13	-0.15	-0.10	0.27	0.54	0.59
Beta-amyloid 42	0.51	0.48	0.23	0.35	0.51	0.56	-0.29	0.73	0.23	0.48	0.64	0.51	0.56
Beta2-microglobulin	0.56	0.24	0.30	0.49	0.48	0.43	0.37	0.73	0.00	0.34	0.37	0.42	0.58
Beta-lactoglobulin	0.26	0.33	0.18	0.44	0.19	0.53	0.19	-0.16	0.11	0.18	0.27	0.39	0.35
Calcitonin	-0.25	-0.51	0.00	-0.23	0.00	-0.48	0.00	-0.54	0.00	0.00	0.45	-0.23	-0.23
Casein	0.06	0.29	0.13	0.27	0.36	0.17	-0.02	-0.22	-0.13	0.00	0.37	0.31	0.19
CspB (*B. subtilis*)	0.11	0.00	0.05	0.08	0.05	0.09	0.23	-0.23	0.05	0.00	-0.05	0.09	0.09
Cystatin C	0.40	0.43	0.66	0.48	0.34	0.42	0.28	0.37	-0.06	-0.06	0.31	0.44	0.50
Myoglobin (horse)	0.42	0.28	0.33	0.25	0.41	0.21	0.46	0.35	0.29	-0.16	0.37	0.30	0.43
Gelsolin	-0.16	0.02	0.07	-0.07	-0.07	0.03	-0.02	-0.05	-0.08	-0.07	0.09	-0.04	-0.06
Het-s (*P. anserina*)	-0.09	0.18	-0.03	-0.22	0.11	-0.04	0.23	0.06	0.04	-0.01	0.24	-0.11	0.10
Insulin (A & B chain)	0.30	0.16	0.16	0.32	0.20	0.14	0.25	0.46	0.30	0.26	0.50	0.36	0.48
Kerato-epithelin	0.06	-0.01	-0.05	0.04	-0.06	0.08	-0.16	0.10	-0.03	0.35	0.16	0.10	0.06
Lactoferrin	0.03	-0.08	-0.03	-0.05	0.13	-0.08	0.11	0.20	0.18	0.19	0.10	0.09	0.08
Lung Surf. Protein C	0.73	0.60	0.27	0.32	0.52	0.00	0.46	0.32	0.68	0.68	0.47	0.32	0.76
Lysozyme C	0.38	0.37	0.00	0.34	0.22	0.17	0.04	0.85	0.09	0.00	0.20	0.30	0.52
Major Prion Protein	0.63	0.34	0.33	0.44	0.55	0.70	0.56	0.45	0.35	0.26	0.32	0.51	0.63
Medin	0.44	0.54	0.00	-0.39	0.84	0.06	0.45	0.70	0.00	0.47	0.32	0.18	0.66
proBNP	1.00	0.31	0.92	0.74	0.93	0.47	0.40	0.50	0.74	0.84	0.92	0.78	0.87
ODAM	0.04	0.12	-0.07	0.31	0.36	0.18	0.24	-0.29	0.00	0.30	0.11	0.33	0.28
Prolactin	0.05	0.15	-0.22	0.26	0.22	0.11	0.29	0.07	0.00	0.22	0.08	0.17	0.22
RepA (*P. syringae*)	0.24	0.30	0.35	0.25	-0.01	0.27	-0.02	-0.17	0.14	0.55	0.04	0.32	0.31
Semenogelin I	0.15	-0.19	-0.12	0.18	0.17	0.08	-0.10	0.04	0.11	0.00	-0.10	0.17	0.10
Serum Amyloid A	0.62	-0.16	-0.09	-0.15	0.00	0.43	-0.10	0.57	0.00	0.74	0.32	0.60	0.60
Sup35 (*S. cerevisiae*)	-0.06	0.11	0.23	-0.04	-0.05	-0.05	-0.08	0.13	-0.03	-0.03	0.06	-0.04	-0.05
Alpha-synuclein	0.51	0.27	0.27	0.00	0.67	0.55	0.47	0.64	0.22	0.32	0.72	0.36	0.64
Tau	0.21	-0.04	0.57	0.26	0.38	0.22	0.28	0.29	0.18	0.00	0.32	0.41	0.50
Transthyretin	0.35	0.41	-0.10	0.33	0.37	0.44	0.17	0.97	0.29	0.30	0.07	0.39	0.44
Ure2p (*S. cerevisiae*)	-0.39	0.23	0.22	-0.30	-0.33	-0.34	-0.24	-0.23	-0.19	-0.16	0.01	-0.20	-0.24
**AVERAGE MCC (PER PROTEIN)**	**0.27**	**0.17**	**0.17**	**0.20**	**0.28**	**0.20**	**0.18**	**0.24**	**0.12**	**0.19**	**0.29**	**0.28**	**0.34**
